# Cigarette Experimentation and the Population Attributable Fraction for Associated Genetic and Non-Genetic Risk Factors

**DOI:** 10.1371/journal.pone.0053868

**Published:** 2013-01-16

**Authors:** Anna V. Wilkinson, Michael D. Swartz, Xiaoying Yu, Margaret R. Spitz, Sanjay Shete

**Affiliations:** 1 University of Texas School of Public Health, Austin Regional Campus, Austin, Texas, United States of America; 2 University of Texas School of Public Health, Houston, Texas, United States of America; 3 Dan L. Duncan Cancer Center, Baylor College of Medicine, Houston, Texas, United States of America; 4 University of Texas M.D. Anderson Center, Division of Quantitative Sciences, Houston, Texas, United States of America; The University of Chicago, United States of America

## Abstract

**Background:**

We, and others, have shown that experimenting with cigarettes is a function of both non-genetic and genetic factors. In this analysis we ask: how much of the total risk of experimenting with cigarettes, among those who had not experimented with cigarettes when they enrolled in a prospective cohort, is attributable to genetic factors and to non-genetic factors?

**Methods:**

Participants (N = 1,118 Mexican origin youth), recruited from a large population-based cohort study in Houston, Texas, provided prospective data on cigarette experimentation over three years. Non-genetic data were elicited twice – baseline and follow-up. Participants were genotyped for 672 functional and tagging variants in the dopamine, serotonin and opioid pathways.

**Results:**

In the overall model, the adjusted combined non-genetic PAF was 71.2% and the adjusted combined genetic PAF was 58.5%. Among committed never smokers the adjusted combined non-genetic PAF was 67.0% and the adjusted combined genetic PAF was 53.5%. However, among cognitively susceptible youth, the adjusted combined non-genetic PAF was 52.0% and the adjusted combined genetic PAF was 68.4%.

**Conclusions:**

Our results suggest there may be differences in genotypes between youth who think they will try cigarettes in the future compared to their peers who think they will not and underscore the possibility that the relative influence of genetic vs. non-genetic factors on the uptake of smoking may vary between these two groups of youth.

**Impact:**

A clearer understanding of the relative role of genetic vs. non-genetic factors in the uptake of smoking may have implications for the design of prevention programs.

## Introduction

Experimenting with cigarettes is a function of both non-genetic and genetic factors [1], . Cognitive susceptibility to smoking, a construct that combines behavioral intentions with peer influence, is one of the strongest and most consistent non-genetic factors associated with experimenting with cigarettes [3]–[5]. Using a prospective cohort study design, we have reported that cognitive susceptibility to smoking predicts future experimentation with cigarettes and modifies the relationship between established non-genetic risk factors associated with experimenting with cigarettes (such as positive outcome expectations, living with a smoker and risk taking tendencies) and experimenting with cigarettes [3]. Subsequently we found six SNPs – three in the dopamine pathway (rs12422191 on *DRD2*, rs10052016 on *SLC6A3*, and rs8119844 on *SNAP25*), two in the serotonin pathway (rs6297 on *HTR1B* and rs9567732 on *HTR2A*), and an opioid receptor variant (rs9322451 on *OPMR1*) – were significantly associated with experimenting with cigarettes [2]. We further noted that cognitive susceptibility to smoking modifies the relationship between these six SNPs and experimenting with cigarettes. Among committed never smokers, three genes (*OPRM1, SNAP25, HTR1B*) were associated with experimentation as were all psychosocial factors. Among susceptible youth older age at baseline, living with a smoker, and three different genes (*HTR2A, DRD2, SLC6A3*) predicted experimentation [2].

In the current analysis we seek to expand our previous work [2] by asking the question: how much of the total risk of experimenting with cigarettes, among those who had not experimented with cigarettes when they enrolled in the prospective cohort is attributable to genetic factors and how much is attributable to non-genetic factors? Specifically we aim to determine the overall proportion of experimenting with cigarettes attributable to non-genetic and genetic factors in a) the overall sample, b) committed never smokers, and c) cognitively susceptible youth. A clearer understanding of the relative role of genetic vs. non-genetic factors in the uptake of smoking may have implications for the design of prevention programs.

## Methods

### Participant Recruitment and Data Collection

Detailed descriptions of the population-based cohort recruitment methodology from where our study participants were drawn [6] have been published. The current study population and baseline data collection procedures also have been published [7] as have the procedures for DNA processing, SNP selection, and genotyping [2]. The current analysis is based on data from the N = 1,118 adolescents recruited in 2005–06 and followed-up, on average 30 months later (SD = 4.8 months), in 2008–09 and reported on in Wilkinson et al [2]. At baseline, after consenting into the study, each participant completed a 5-minute personal interview during which demographic data were collected. The remainder of the survey was completed on a personal digital assistant (PDA). All participants provided buccal samples at baseline. The institutional review board at M. D. Anderson Cancer Center approved all aspects of this study.

### Measures

To briefly review the non-genetic variables included in the current analysis, our primary outcome of interest is new experimentation assessed by two items from the Youth Risk Behavior Survey [8]: “Have you ever smoked a whole cigarette?” and “Have you ever tried a cigarette, even a puff?” We defined new experimentation as anyone who answered “no” to both questions at baseline, but “yes” to either question at any follow-up interview. Non-genetic covariates used in the analysis were all assessed at baseline. These included cognitive susceptibility to smoking [5], risk taking behaviors, household social influence (i.e. the number of smoking household members residing with participant), and positive outcome expectations [9]. All analyses adjusted for gender and age as both are associated with cigarette experimentation.

### Statistical Analysis

Using new experimentation vs. no experimentation as our outcome and excluding all individuals who had experimented at baseline, we compute the population attributable fraction (PAF) for each factor. The PAF provides an estimate of the percentage of overall risk in a population due to a specific risk factor [10]. We first fit three multiple logistic regression models (one overall model and two stratified on cognitive susceptibility status). The logistic regression framework allows us to account for confounders and thus, allows a more accurate estimation of the PAF. We adjusted for ethnic variation using the same principal components analyses for the genotyping data and using the top two eigenvectors as covariates in the logistic regression models we previously used [2]. SNPs that exhibited a protective effect were reverse coded. Next we followed Greenland and Drescher’s method for computing the PAF using logistic models with cohort data [11]. Briefly, we used the maximum likelihood estimators derived by Greenland and Drescher [11] for cohort data to estimate the PAF and the variance of the estimates to make inference on the PAF. The maximum likelihood estimators they developed were based on the maximizing the logistic regression likelihood using the total cases in a cohort compared to the total number exposed in the cohort. We estimated the PAF and its 95% CI for each SNP and each non-genetic risk factor, adjusted for all other variables in the model. And finally, we estimated the PAF for all genetic and non-genetic risk factors, again adjusting for all other variables. We computed the % overlap in PAF as the difference between each model (genetic or non-genetic PAF) and the total PAF, similar to Shikata et al [12]. We repeated the analyses in subsets of the participants stratified by cognitive susceptibility. However, in the subgroup analyses of susceptible youth, the events per predictor variable (EPV) in our data are 7 which is less than the suggested value of 10 EPV [13]. However, based on a Monte Carlo study, Vittinghoff and McCulloch [14] concluded that the issues such as confidence interval coverage less than 93%, type 1 error greater than 7%, or relative bias greater than 15% is uncommon with 5–9 EPV. Furthermore, they found that bootstrap confidence intervals were more conservative than Wald confidence intervals, often with coverage greater than 95%. Therefore, we computed the 95% CIs of the estimates of the PAFs in the stratified analyses by cognitive susceptibility using bootstrap resampling [15]. Specifically, we obtained 1000 random samples by resampling from the original dataset, with replacement and each sample had the same size (n = 1,118) as the original sample. The PAFs were calculated for each bootstrap sample to construct the empirical distribution of the PAFs. Then we used the bootstrap quantiles to construct an empirical 95% confidence interval from the distribution. All computations were completed in STATA 12, and PAFs were computed using the STATA command Punaf [16].

## Results

In table 1 we summarize the distribution of the non-genetic and genetic risk factors by experimenter status reported in the original study [2] and present the adjusted attributable fractions and 95% confidence intervals in tables 2 and 3. Of the 211 (18.9%) participants who began experimenting over the course of the study, 62.6% were male compared with 44.3% for never experimenters (p<0.001). Experimenters were significantly more likely to be 13 at baseline (42.7% vs. 21.3%; p<0.001) and live with at least one smoker (55.4% vs. 35.3%; p<0.001). A higher proportion of experimenters held positive outcome expectations about smoking (56.9% vs. 34.6%; p<0.001), reported risk taking tendencies (70.1% vs. 50.5%; p<0.001), and being cognitively susceptible to smoking (43.6% vs. 17.0%) than never experimenters (Table 1).

**Table 1:** Distribution of demographic characteristics, non-genetic and genetic covariates by new experimenter status (N = 1,118).

		Experimenter Status		
		New Experimenter	Never Experimenter	
	N (%)	N (%)	N (%)	p-value
Overall	1,118 (100.0)	211 (18.9)	907 (81.1)	
**Non-genetic Covariates**				
Gender				
Females*	584 (52.2)	79 (37.4)	505 (55.7)	
Males	534 (47.8)	132 (62.6)	402 (44.3)	<0.001
Age at baseline (years)				
11 or 12*	835 (74.7)	121 (57.3)	714 (78.7)	
13	283 (25.3)	90 (42.7)	193 (21.3)	<0.001
Mean (SD)	11.89 (0.84)	12.18 (0.87)	11.75 (0.80)	<0.001
No. of household members who smoke				
None*	681 (60.9)	94 (44.6)	587 (64.7)	
One	341 (30.5)	84 (39.8)	257 (28.4)	
≥ Two	96 (8.6)	33 (15.6)	63 (6.9)	<0.001
Mean (SD)	0.55 (0.76)	0.75 (0.84)	0.43 (0.66)	<0.001
Risk taking				
Averse*	512 (45.8)	63 (29.9)	449 (49.5)	
Some	606 (54.2)	148 (70.1)	458 (50.5)	<0.001
Mean (SD)	1.68 (0.81)	1.95 (0.90)	1.58 (0.76)	<0.001
Outcome expectations				
None*	684 (61.2)	91 (43.1)	593 (65.4)	
Some	434 (38.8)	120 (56.9)	314 (34.6)	<0.001
Mean (SD)	1.26 (0.40)	1.35 (0.42)	1.19 (0.35)	<0.001
Susceptible at baseline				
No*	872 (78.0)	119 (56.4)	753 (83.0)	
Yes	246 (22.0)	92 (43.6)	154 (17.0)	<0.001
**Genetic Covariates**				
OPRM1 (rs9322451)				
0	822 (73.5)	174 (21.2)	648 (78.8)	
1*	296 (26.5)	37 (12.5)	259 (87.5)	0.001
HTR1B (rs6297)				
0*	990 (88.6)	117 (17.9)	813 (82.1)	
1	128 (11.4)	34 (26.6)	94 (73.4)	0.018
SNAP25 (rs8119844)				
0*	1,089 (97.4)	200 (18.4)	889 (81.6)	
1	29 (2.6)	11 (37.9)	18 (62.1)	0.008
SLC6A3 (rs10052016)				
0	620 (55.5)	133 (21.5)	487 (78.5)	
1*	498 (44.5)	78 (15.7)	420 (84.3)	0.014
HTR2A (rs9567732)				
0	530 (47.7)	117 (22.1)	413 (77.9)	
1*	588 (52.6)	94 (16.0)	494 (84.0)	0.009
DRD2 (rs12422191)				
0*	980 (87.7)	175 (17.9)	805 (82.1)	
1	138 (12.3)	36 (26.1)	102 (73.9)	0.021

* Reference group used for PAF and logistic computations.

**Table 2:** Population attributable fraction (PAF) after logistic regression (N = 1,118).

	Psychosocial and genetic factors		
	PAF	95% CI	Reference Group *
**Non-genetic Covariates**			
Cognitively susceptible	0.183	0.101–0.258	Not susceptible
Age 13 at baseline	0.167	0.088–0.240	11 or 12 years old
Male	0.225	0.106–0.328	Female
Outcome expectations	0.091	0.008–0.167	None
Household membersmokes	0.160	0.073–0.239	None
Risk taking tendencies	0.134	0.026–0.229	Risk averse
** All psychosocial factors**	**0.712**	**0.614–0.785**	
**Genetic Covariates (SNPs)**			
SNAP25 (rs8119844)	0.029	0.007–0.051	Homozygous major allele
OPRM1 (rs9322451)	0.289	0.092–0.444	1
HTR1B (rs6297)	0.054	0.009–0.097	0
SLC6A3 (rs10052016)	0.163	0.034–0.276	1
HTR2A (rs9567732)	0.115	−0.0001–0.217	1
DRD2 (rs12422191)	0.063	0.018–0.106	0
** All genetic factors**	**0.585**	**0.393–0.716**	

* Reference group also identified in table 1.

**Table 3:** Population attributable fraction (PAF) after logistic regression for new experimentation stratified by cognitive susceptibility status (N = 1,118).

	Committed Never Smokers (N = 872)		Susceptibles (N = 246)	
	PAF	95% CI	PAF	95% CI
**Non-genetic Covariates**				
Age 13 at baseline	0.146	0.051–0.248	0.203	0.079–0.320
Male	0.267	0.120–0.417	0.136	−0.051–0.312
Outcome expectations	0.150	0.068–0.237	−0.017	−0.157–0.140
Household member smokes	0.162	0.056–0.257	0.160	0.035–0.284
Risk taking tendencies	0.185	0.059–0.305	0.076	−0.102–0.255
** All psychosocial factors**	**0.670**	**0.539–0.782**	**0.520**	**0.253–0.733**
**Genetic Covariates (SNPs)**				
SNAP25 (rs8119844)	0.050	0.012–0.091	0.007	−0.005–0.028
OPRM1 (rs9322451)	0.319	0.055–0.554	0.214	−0.061–0.477
HTR1B (rs6297)	0.078	0.010–0.148	0.021	−0.036–0.079
SLC6A3 (rs10052016)	0.073	−0.106–0.249	0.258	0.100–0.419
HTR2A (rs9567732)	0.081	−0.068–0.237	0.190	0.034–0.353
DRD2 (rs12422191)	0.036	−0.025–0.108	0.094	0.042–0.161
** All genetic factors**	**0.535**	**0.251–0.759**	**0.684**	**0.437–0.857**

In the overall model, the adjusted combined non-genetic PAF was 71.2% and the adjusted combined genetic PAF was 58.5% (Table 2). In this model, the PAF was greatest for *OPRM1* (28.9%), being male (22.5%), and cognitively susceptible to smoking (18.3%). In the models stratified on cognitive susceptibility (Table 3), among the committed never smokers the adjusted combined non-genetic PAF was 67.0% and the adjusted combined genetic PAF was 53.5%. Again, the PAF was greatest for *OPRM1* (31.9%) and being male (26.7%). However, among the cognitively susceptible, the adjusted combined non-genetic PAF was 52.0% and the adjusted combined genetic PAF was 68.4%. In this model, the PAF was greatest for *SLC6A3* (25.8%) and older age at baseline (20.3%).

**Figure 1:**
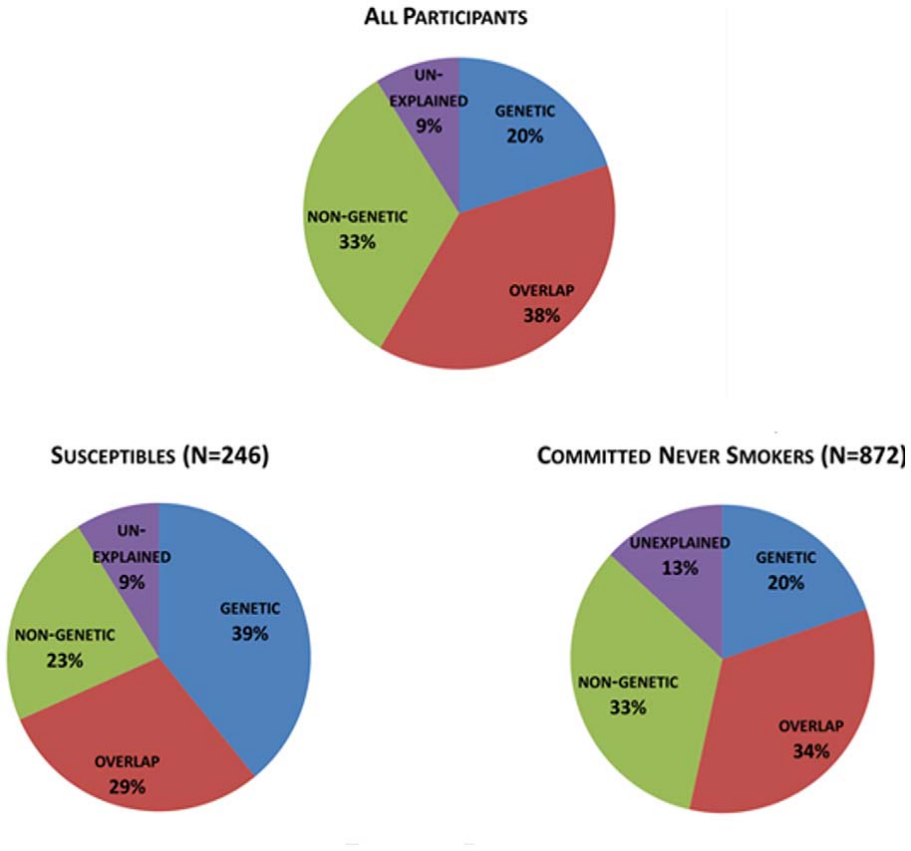
Estimated PAFs for non-genetic and genetic risk factors associated with cigarette experimentation, overall and by cognitive susceptibility status.

In Figure 1 we present the estimated PAF, overall and by cognitive susceptibility status, for the non-genetic and the genetic risk factors alone, as well as the PAF due to the presence of both genetic and non-genetic risk factors, and the proportion that was not explained by either group of risk factors. Overall we found that 33% was due to non-genetic factors alone, 20% due to genetic factors alone, 34% due to the presence of both the non-genetic and genetic risk factors examined, and 9% was unexplained. Among the committed never smokers 33% was due to non-genetic factors alone, 20% due to genetic factors alone, 38% due to the presence of both the non-genetic and genetic risk factors examined, and 13% was unexplained. Among the youth who are susceptible to smoking 23% was due to non-genetic factors, 39% due to genetic factors, 29% due to the presence of both the non-genetic and genetic risk factors examined, and 9% was unexplained.

## Discussion

In our study we obtained estimates of PAF for genetic and non-genetic risk factors associated with experimenting with cigarettes using a longitudinal prospective cohort study. Thus, our study design afforded the opportunity to examine incident cases of experimenting with cigarettes rather than prevalent cases. In the overall analysis we found that the influence of the non-genetic factors assessed at baseline accounted for a higher proportion of the risk associated with new experimentation compared to the genetic factors. Overall, 71% of the risk associated with smoking initiation was attributable to the non-genetic factors and 59% to the genes. It should be noted that our participants did report significant changes in the non-genetic risk factors (perceived positive outcome expectations, number of people who smoked in the household and risk taking tendencies) during the 30-month study period. In a cross-sectional analysis using all variables assessed 30 months after baseline, the association between each of these three variables and being a new experimenter remains significant while controlling for age, gender and the six SNPs (data not shown), which speaks to the stability of the modifiable risk factors during mid to late adolescence.

In addition, we calculated the PAF for both genetic and non-genetic risk factors based on the baseline cognitive susceptibility status when the youth enrolled in the study. Of note, at follow-up, rates of experimentation were three times higher among the cognitively susceptible youth compared to the committed never smokers; 45% of the cognitively susceptible youth reported experimentation during the 30 month follow-up period compared to 15% of the committed never smokers [3]. Among the committed never smokers we found that 67% (95% CI: 53 to 77%) of the risk associated with new experimentation was attributable to non-genetic factors and 54% (95% CI: 22 to 73%) to the genes. However, the relative influence of genes and environment among the cognitively susceptible was reversed, among whom we found 52% (95% CI: 25 to 69%) of the risk attributable to the non-genetic factors and 68% (95% CI: 41 to 83%) to the genes.

The PAF provides an estimate expressed as a percent of the overall risk in a population that is due to the risk factors in question [10]. Thus, we estimated the genetic, the non-genetic, the overlap (i.e. PAF due to the presence of both genetic and non-genetic risk factors), and the unexplained PAF based on baseline cognitive susceptibility status. Among those susceptible to smoking, the PAF due to the presence of both genetic and non-genetic risk factors for experimentation was 29%, and 23% of experimenting with cigarettes was attributable to non-genetic risk factors alone, whereas among the committed never smokers the PAF due to the presence of both genetic and non-genetic risk factors was 34% and 33% was attributable to non-genetic risk factors alone (see Figure 1). In terms of intervention design, which at the moment can only focus on the modifiable non-genetic risk factors, our results underscore the continued need to refine our understanding of these modifiable risk factors, especially among youth who report a cognitive susceptibility to smoking. These youth are not only more likely to experiment with cigarettes compared to their peers who are committed never smokers, but also the modifiable risk factors contribute less to their behavior compared to the committed never smokers.

In the current study, we were interested in experimenting with cigarettes, the first step in the smoking trajectory. Accordingly, the candidate SNPs were selected based on their association with sensation seeking tendencies and risk taking behavior because both are associated with smoking initiation [17]–[19]. On the other hand, the non-genetic risk factors included are those that we, and others, have found to be associated with cigarette experimentation among youth in general [1]. Thus the relative magnitude of attributable risk from the genetic vs. the non-genetic factors could be different had we examined different SNPs and other non-genetic risk factors associated with smoking.

Our study has several strengths. The prospective design allowed us to examine incident experimentation reported during follow-up among participants who had not experimented at baseline. This is important because experimentation with cigarettes is the first step in the uptake of smoking. Further, participants were recruited from a population-based cohort [6], included roughly equal numbers of females and males, and represent a large ethnically homogenous and predominantly low-income sample of Mexican origin youth, an understudied population. The non-genetic risk factors were assessed using validated measures, and all data were collected in the home using personal digital assistants to ensure participant privacy and quality of the data. A final strength is the high retention rate: 87% of the youth provided data on all five contacts.

Conversely, the main limitation of this study is the lack of an independent replication sample; thus we must consider our findings preliminary. In addition, although we examined a large number of SNPs, we did adjust the significance level for each SNP included in this analysis using a BFDP approach [20]. We took a candidate gene pathway approach, and it is possible the pathways we did not examine also contribute to the risk of smoking. For example, the results from a recent genome-wide association study identified two SNPS located in gene desert regions associated with smoking initiation [21]. A third limitation is that participants were all of Mexican origin, and results may not generalize to other ethnicities. Finally, while we did not use salivary cotinine to biochemically validate the participants’ smoking status, we informed participants during the consent process that they might be selected to provide a saliva sample to check their smoking status; this “bogus pipeline” procedure has been shown to increase the validity of self-reported smoking status [22].

### Conclusion

Our results need independent validation, which is not always feasible when studying minority populations, and underscore the possibility that there are differences in genotype between youth who think they will try cigarettes in the future compared to their peers who think they will not try cigarettes. Our results further underscore the possibility that the relative influence of genetic vs. non-genetic factors on the uptake of smoking may vary between these two groups of youth. While we need to be cautious when interpreting the attributable fraction in terms of intervention design [23] and how the results might generalize to youth of other ethnicities, overall, we found that 22% of our sample of Mexican origin youth think they will try cigarettes in the future and for these youth, the relative influence of genetic risk vs. non-genetic risk factors on smoking experimentation appears to be greater compared to youth who think they will not try cigarettes in the future.
